# Application of Membrane Materials in Bioseparation and Downstream Processing

**DOI:** 10.3390/membranes16010046

**Published:** 2026-01-19

**Authors:** Wei Zhang, Lingxue Kong

**Affiliations:** 1 Bioprocessing Technology Institute (BTI), Agency for Science, Technology and Research (A*STAR), 20 Biopolis Way, #06-01 Centros, Singapore 138668, Singapore; 2 Institute for Frontier Materials, Deakin University, Waurn Ponds, Geelong, VIC 3216, Australia

## 1. Introduction

Membrane technologies have played an instrumental role in bioseparation and downstream processing. Membrane operations such as microfiltration, ultrafiltration, and virus filtration are key tools for clarification, concentration, buffer exchange, and viral clearance in biomanufacturing, as they offer mild, selective, and efficient separations for delicate biological products.

With advances in biotherapeutics development, membrane technologies have become increasingly pivotal in bioseparation and downstream processing. Compared with conventional packed-bed chromatography, a major advantage of membrane technologies is their convective mass transfer, which enables faster processing times and higher productivity. This advantage is particularly crucial for processing increasingly complex and fragile large biomolecules, such as bispecific antibodies, viruses, viral vectors, extracellular vesicles, and even cells.

In this Special Issue, Application of Membrane Materials in Bioseparation and Downstream Processing, published in Membranes, we have collected a diverse yet cohesive set of papers that demonstrate advances in the application of membrane technologies in bioseparation and downstream processing. These studies address highly relevant challenges ranging from conventional applications to emerging modalities, and from process optimization to novel membrane material design. Collectively, they underscore the pivotal role of membrane technologies in enabling efficient and robust separations in modern biomanufacturing.

## 2. Overview of Special Issue Contributions

To begin, several papers included in this Special Issue demonstrate advances in conventional applications of membrane technologies in bioseparation and downstream processing, including bioreactor clarification and virus filtration. Mostafavi et al. explore the potential to reduce membrane fouling by integrating a hydrocyclone as the primary clarification operation and investigate the impact of membrane morphology and pore size of integrated tangential flow filtration on overall clarification performance, offering insights that can improve throughput and robustness in early downstream processing stages. Isu et al. investigate the effectiveness of different membrane adsorbers in disrupting reversible aggregates that form during virus removal filtration and contribute to flux decline, thereby helping to guide the development of more effective virus filtration processes for monoclonal antibody production. Afzal et al. examine the effects of proteins on virus retention across different virus removal filters, providing important insights into the development and application of virus filtration in bioprocessing.

Secondly, this Special Issue also showcases novel membrane applications for more complex biological products, including bispecific antibodies, outer membrane vesicles, viral vectors, and cells. Zhao et al. evaluate the impact of the operating buffer system of loading samples on aggregate removal using hydrophobic interaction membrane chromatography, demonstrating that this technique operated in flow-through mode is an effective and robust approach for reducing aggregates during bispecific antibody purification. Li et al. develop a membrane filtration method to isolate *Escherichia coli* Nissle 1917–derived outer membrane vesicles of varying sizes, providing direct experimental evidence and deeper biological insights that outer membrane vesicles of different sizes exhibit heterogeneous properties. Sarmah and Husson demonstrate the feasibility of separating full and empty capsids of adeno-associated virus via ultrafiltration, directly addressing a long-standing bottleneck in viral vector downstream processing and offering a potential alternative solution. Schwarz et al. characterize membrane fouling during the separation of *Vibrio natriegens* biomass from culture broth using a cross-flow filtration plant with ceramic membranes, providing guidance for the design of economical filtration plants and strengthening the potential of establishing *V. natriegens* as a production host for large-scale industrial processes.

Lastly, other contributions included in this Special Issue also summarize recent breakthroughs in rational membrane design and shed light on future directions in the field. Boztepe et al. highlight the growing relevance of ion-exchange nanofibrous membranes in membrane chromatography for protein purification, underscore the importance of computational modelling as a viable predictive approach to guide membrane design and performance prediction and emphasize that integrating AI-guided design with high-throughput characterization could represent a future direction for membrane material innovation.

## 3. Conclusions

In summary, this Special Issue presents a diverse collection of papers on membrane materials in bioseparation and downstream processing, covering applications ranging from conventional bioreactor clarification and viral clearance to the purification of emerging modalities, including bispecific antibodies, outer membrane vesicles, viral vectors, and cells. Collectively, these studies demonstrate that membrane technologies have progressed from an auxiliary role to a central, enabling tool in biomanufacturing. We hope this Special Issue not only provides a timely update on the applications of membrane materials in bioseparation and downstream processing but also serves as a catalyst for continued innovation in membrane technologies.

